# The Role of Brain-Derived Neurotrophic Factor as an Essential Mediator in Neuronal Functions and the Therapeutic Potential of Its Mimetics for Neuroprotection in Neurologic and Psychiatric Disorders

**DOI:** 10.3390/molecules30040848

**Published:** 2025-02-12

**Authors:** Tadahiro Numakawa, Ryutaro Kajihara

**Affiliations:** 1 Department of Cell Modulation, Institute of Molecular Embryology and Genetics, Kumamoto University, Kumamoto 860-0811, Japan; 2 Department of Hematology and Immunology, Faculty of Life Science, Kumamoto University, Kumamoto 862-0976, Japan

**Keywords:** BDNF, TrkB, depression, schizophrenia, Alzheimer’s disease, Parkinson’s disease, Huntington’s disease, BDNF mimetics

## Abstract

Among neurotrophins, including nerve growth factor (NGF), brain-derived neurotrophic factor (BDNF), neurotrophin-3 (NT-3), and neurotrophin-4 (NT-4/5), BDNF has been extensively studied for its physiological role in cell survival and synaptic regulation in the central nervous system’s (CNS’s) neurons. BDNF binds to TrkB (a tyrosine kinase) with high affinity, and the resulting downstream intracellular signaling cascades play crucial roles in determining cell fate, including neuronal differentiation and maturation of the CNS neurons. It has been well demonstrated that the downregulation/dysregulation of the BDNF/TrkB system is implicated in the pathogenesis of neurologic and psychiatric disorders, such as Alzheimer’s disease (AD) and depression. Interestingly, the effects of BDNF mimetic compounds including flavonoids, small molecules which can activate TrkB-mediated signaling, have been extensively investigated as potential therapeutic strategies for brain diseases, given that p75NTR, a common neurotrophin receptor, also contributes to cell death under a variety of pathological conditions such as neurodegeneration. Since the downregulation of the BDNF/TrkB system is associated with the pathophysiology of neurodegenerative diseases and psychiatric disorders, understanding how alterations in the BDNF/TrkB system contribute to disease progression could provide valuable insight for the prevention of these brain diseases. The present review shows recent advances in the molecular mechanisms underlying the BDNF/TrkB system in neuronal survival and plasticity, providing critical insights into the potential therapeutic impact of BDNF mimetics in the pathophysiology of brain diseases.

## 1. Introduction

A growing body of evidence has demonstrated the critical importance of BDNF and its receptor in the development and function of brains. BDNF, which belongs to neurotrophins, has multiple roles in both the PNS and CNS neurons [1,2,3,4]. Trks, tyrosine protein kinase receptors for neurotrophins, consist of TrkA, TrkB, and TrkC, and mainly regulate the cell proliferation, differentiation, maturation, and synaptic function in the PNS and CNS neurons when specific neurotrophins bind to them [3,4]. It is well known that NGF, the first identified neurotrophin, binds to TrkA with high affinity. BDNF, which is expressed in a variety of brain regions including the cerebral cortex and hippocampus, contributing to learning and memory function in the brain, also has a high-affinity TrkB receptor [1,2,5,6]. Similarly, NT-3 binds to TrkC, and NT-4 binds to TrkB, although all the members of the neurotrophin family interact with the p75 neurotrophin receptor (p75NTR), which has no catalytic activity, with low affinity [7].

**Figure 1:**
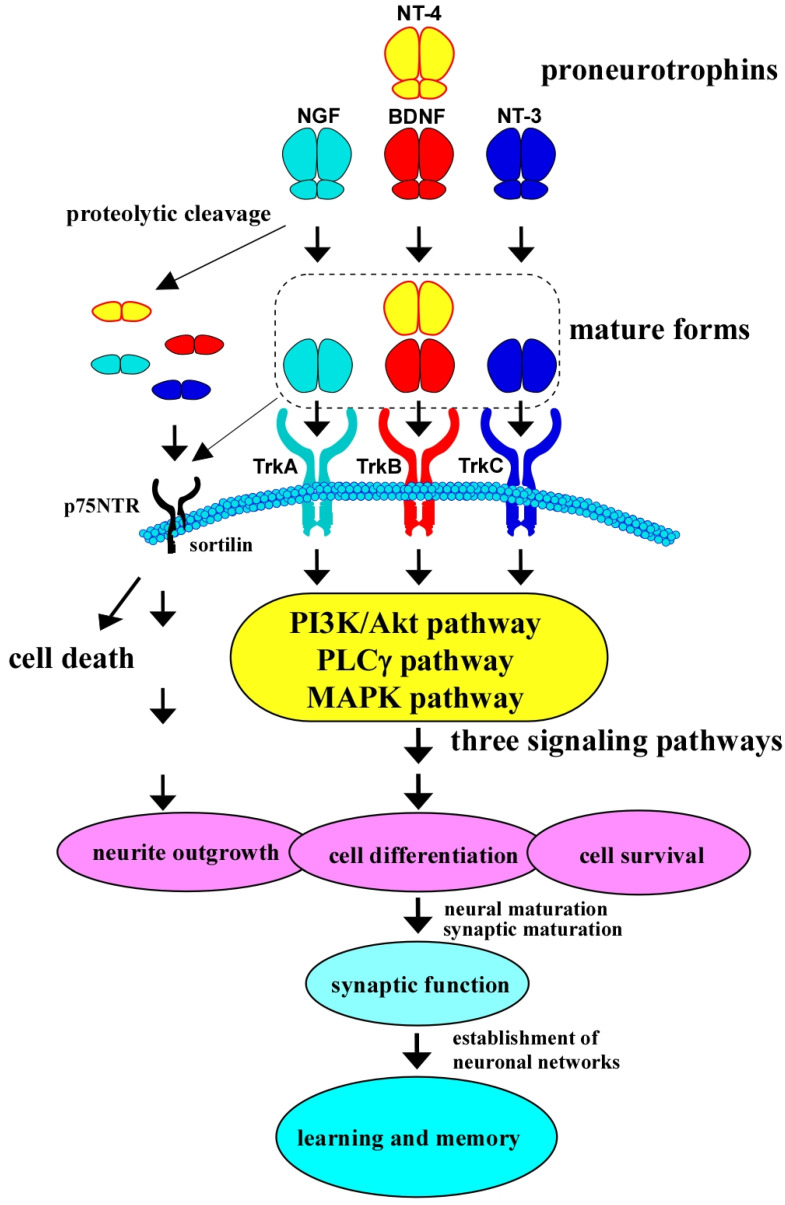
Neurotrophins and their receptors in the CNS neurons. Brain-derived neurotrophic factor (BDNF), nerve growth factor (NGF, the first identified one), neurotrophin-3 (NT-3), and neurotrophin-4 (NT-4) are produced first as precursor pro-forms (proneurotrophins). Proneurotrophins bind to p75NTR (a common receptor for all neurotrophins) with a high affinity. Sortilin, a co-receptor, is also critical for the binding with pro-forms, resulting in neuronal cell death. After receiving proteolytic cleavage, mature BDNF produced from pro-forms binds to TrkB, a high affinity receptor for mature BDNF and NT-4, and triggers downstream intracellular signaling, including the MAPK, PLCγ, and PI3K/Akt pathways, leading to cell differentiation and survival, in addition to neurite outgrowth. It is well known that the BDNF/TrkB system, which has multiple roles in neural cell aspects, is closely associated with synaptic function and learning and memory function in the brain. NGF binds to TrkA with a high affinity; similarly, NT-3 binds to TrkC.

Firstly, all members of the neurotrophins, including BDNF, are translated as precursors (named proneurotrophins), which bind to the p75NTR with high affinity, leading to apoptosis and negatively regulating neuronal growth and synaptic plasticity [8,9,10]. On the other hand, small mature neurotrophins preferentially interact with Trks after receiving the subsequent cleaving process (mature NGF, mature BDNF, and so on; see Figure 1) [5,7].

Importantly, evidence indicates the critical contribution of BDNF in neurogenesis, synaptic plasticity, and neuronal survival, which are essential for cognitive function, emotional regulation, and resilience to stress. The roles of BDNF in the CNS are vital for brain function; thus, dysregulation of BDNF function has been implicated in the cognitive impairment observed in the various mental illnesses, including depression [5]. It has been demonstrated that altered expressions of BDNF and/or TrkB are associated with major depressive disorder (MDD) [11]. Additionally, evidence suggests that changes in intracellular signaling via activation of receptors, including TrkB and p75NTR, are involved in the pathogenesis of depression [12]. Furthermore, overactivation of hypothalamic–pituitary–adrenal (HPA) axis is often influenced by environmental challenges, leading to abnormally elevated levels of glucocorticoids, which in turn affect the BDNF/TrkB system [13,14]. Interestingly, altered BDNF function has also been suggested to play a role in schizophrenia, a disorder characterized by abnormalities in thinking and emotions [15]. Moreover, it was revealed that schizophrenia-like behaviors observed in Bdnf-e6-/- mice (promoter VI mutant mice) exposed to postnatal stress improved after treatment with an antagonist for glucocorticoids or an agonistic antibody for TrkB, suggesting the involvement of both stress and the BDNF/TrkB system in the pathogenesis of schizophrenia [16]. Therefore, it has been demonstrated that upregulation of the BDNF/TrkB system is effective against the cognitive impairment of mental illnesses [6,17]. In this review, we discuss current studies concerning the upregulation of BDNF by natural compounds and the influence of BDNF mimetics in behaviors of in vivo and in vitro models for mental illnesses.

AD, which is recognized as the most common neurodegenerative disorder, shows abnormal accumulation of amyloid-beta (Aβ) and neurofibrillary tangles in which hyperphosphorylated tau protein is aggregated in the brain tissue, and in which the patient exhibits significant cognitive decline. A variety of pharmacological interventions aiming at the deprivation of Aβ and tau proteins have carried out; however, there has been limited success in treatment for progression of the disease [18]. Recently, as one of the alternative therapeutic targets going over the amyloid-centric approach, facilitation of the BDNF/TrkB system has been considered a critical target for delaying disease progression in AD [19,20,21,22]. Patients with Parkinson’s disease (PD), which is also a neurodegenerative disorder, display bradykinesia, rigidity, and tremors, in addition to non-motor symptoms impacting quality of life, and exhibit the loss of dopaminergic neurons within the substantia nigra pars compacta, resulting in depletion of striatal dopamine levels. In PD pathogenesis, it has also been demonstrated that the downregulation of the BDNF/TrkB system could contribute to dopaminergic neuronal degeneration [23]. Taken together, how to enhance the BDNF/TrkB system is a promising target for therapeutic development in these brain diseases. Similarly, it has been demonstrated that changed neurotrophic support by BDNF/TrkB is involved in the pathogenesis of Huntington’s disease (HD), which exhibits significant neurodegeneration, particularly in the striatum and cortex [24].

Recent evidence has demonstrated that natural compounds which increase the endogenous expression of BDNF and/or TrkB, as well as BDNF mimetic compounds that are small molecules passing the blood–brain barrier (BBB), look promising to treat brain diseases including mental and neurological disorders. Here, we show recent evidence in regulation of the BDNF/TrkB system by small molecules using in vivo and in vitro disease models, to take critical insights for developing the potential therapeutic approach to mental and neurological disorders.

## 2. Biological Roles of BDNF/TrkB System and Its Downstream Intracellular Signaling

There are three main intracellular signaling pathways triggered after TrkB activation. BDNF can stimulate the phospholipase Cγ (PLCγ), which is involved in Ca^2+^ homeostasis regulated by BDNF, the mitogen-activated protein kinase (MAPK) contributing to a variety of neuronal aspects including synaptic plasticity, and the phosphatidylinositol 3 kinase (PI3K)/Akt maintaining cell survival [25,26,27].

We previously reported that enhanced intracellular Ca^2+^ concentration by BDNF is important for release of glutamate, an excitatory ammino acid transmitter, which is mainly dependent on the PLCγ pathway [28]. Furthermore, we also found a significant downregulation of the PLCγ pathway in brain neurons obtained from pups with low birth weight produced by intrauterine growth retardation (IUGR) [29]. Importantly, it is well accepted that the activation of Ca^2+^-calmodulin-dependent kinases (CaMKs) and protein kinase C and the generation of 1,2-diacylglycerol are regulated via activation of the PLCγ pathway [26,30].

The family of MAPK, which is a serine–threonine kinase, is composed of p38, c-Jun NH2-terminal kinase (JNK, or stress-activated protein kinase), and ERK, and has multiple roles in mammalian cells. In many kinds of cell populations, including the CNS neurons, MAPKs contribute to cell proliferation, differentiation, survival, maturation, and synaptic function [31,32,33]. In particular, ERK regulates the expression of synaptic proteins stimulated by BDNF. We previously reported that attenuated ERK levels after glucocorticoid (stress hormone) exposure resulted in downregulation of the BDNF-enhanced expression of synaptic proteins [34]. Interestingly, recent evidence suggests that SPROUTY2, a critical regulatory molecule for the signaling of neurotrophic factors including BDNF, is possibly an effective therapeutic target for neurodevelopmental disorders because it regulates a variety of neuronal aspects, including cell death and axon outgrowth, through affecting the ERK signaling pathway (see [35]).

Importantly, the mammalian target of rapamycin (mTOR), which is a key molecule for cellular homeostasis, functions as a downstream molecule of the PI3K/Akt signaling pathway, leading to facilitating cell differentiation and survival [36,37,38,39]; therefore, the PI3K/Akt/mTOR pathway also exerts a critical role in BDNF/TrkB-mediated neuronal survival in the CNS neurons. Furthermore, evidence demonstrates that the Akt/cyclic adenosine monophosphate (cAMP) response element-binding protein (CREB) pathway, which induces the upregulation of BDNF, is a promising therapeutic target in neurodegenerative diseases including AD [40]. In addition to ERK signaling, these PI3K/Akt/mTOR and Akt/CREB pathways are also suggested to be involved in the regulation of the apoptosis observed in neurodegenerative diseases [41].

## 3. BDNF/TrkB System and Antidepressant Effects of Natural Compounds in Depression Models

As mentioned above, a growing body of evidence has demonstrated that altered expression and function of the BDNF/TrkB system are closely correlated with major depressive disorder (MDD) [11]. At the current time, although the pathogenesis of depression is not fully understood, it has been suggested that signal pathways affected by BDNF and its receptors (TrkB and p75NTR) have a role in the pathogenesis of depression [12]. Since patients with depressive disorders exhibit cognitive impairments [42], decreased activation of the BDNF/TrkB system, which regulates neuronal function, has been considered as one of the major mechanisms underlying the pathophysiology of depressive disorders [13]. Importantly, it is well known that the overactivation of the hypothalamic–pituitary–adrenal (HPA) axis induces abnormally increased glucocorticoids (the stress hormones), which influence the BDNF/TrkB system [13]. A variety of environmental challenges affect the status of the HPA axis, leading to changed blood levels of glucocorticoids, which in turn influence synaptic function and BDNF-mediated neuronal aspects [13,14].

When searching novel antidepressant drugs, Bupleurum, which is the traditional Chinese medicine to treat liver illness, including its inflammation, fibrosis, and cancer, is a promising candidate [43]. It has been shown that rutin, puerarin, saikosaponin A, saikosaponin D, and quercetin, which are ingredients obtained from Bupleurum, have antidepressant effects through different mechanisms including the regulation of neurotransmitters, the NMDA receptor system, and the BDNF/TrkB system [43]. The 18β-glycyrrhetinic acid (18β-GA) in Xiaoyaosan (XYS), which is a traditional Chinese medicine and used for the treatment of depression, has been identified as a primary active compound [44]. Using the chronic social defeat stress (CSDS) model, it was revealed that an injection of XYS showed antidepressant-like effects with the activation of BDNF transcription in the medial prefrontal cortex of CSDS mice. Furthermore, 18β-GA was identified as the primary product in the mouse brain after XYS administration, which is also found to interact with ERK, leading to the activation of ERK, and to induce BDNF transcription by activating nuclear factor-erythroid factor 2-related factor 2 (Nrf2), CREB, and c-Jun [44]. Many studies show neuroprotective properties of phytocannabinoids and a relationship between cannabis and psychiatric conditions, including depression, has been discussed [45]. Interestingly, a recent study examined the biological effect of both natural tetrahydro-cannabiorcol (trans-(−)-Δ9-THCO) and unnatural Δ9-tetrahydrocannabiorcol (trans-(+)-Δ9-THCO), focusing on the levels of BDNF as an indicator for potential in neuropathologies [46]. Both THCO enantiomers significantly reversed cell death caused by CORT (corticosterone), one of the glucocorticoids, in the SH-SY5Y cells, and increased the expression of BDNF simultaneously. It was also shown that unnatural trans-(+)-Δ9-THCO has better activity compared with that of natural enantiomer [46]. It has been demonstrated that the nuclear factor erythroid 2-related factor 2-antioxidant responsive element (Nrf2-ARE) is involved in cellular defense systems under oxidative stress [47]. Interestingly, the activation and/or nuclear translocation of Nrf2 are triggered via the PI3K/Akt pathway stimulated by the BDNF/TrkB system. Notably, recent evidence shows that indole-3-carbinol and its metabolite, diindolylmethane, exert neuroprotective effects via activating Akt, leading to antioxidant action, by inhibiting the Nrf2-kelch-like ECH-associated protein 1 (Keap1) complex [47]. Recently, NMC-4 (new macamide compound-4) has been synthesized and analyzed [48]. Using a rat model of depression caused by chronic unpredictable mild stress, the antidepressant action of NMC-4 significantly normalized the reduced BDNF/PI3K/Akt pathways in the cerebral cortex of depressed rats. Furthermore, increased levels of IL-1β, IL-6, and TNF-α in the cortex of depressed animals were all downregulated by NMC-4 application [48]. Recently, the antidepressant effect of ononin, which is a flavonoid compound, was investigated using depressive rat models induced by chronic mild stress (CMS) [49]. It was revealed that significantly decreased depression-like behaviors assessed with a sucrose consumption test, open field test, and tail suspension test (TST) were achieved after ononin treatment [49]. Markedly, the treatment with ononin increased the protein levels of neurotrophic factors including BDNF, GDNF, and NGF in the hippocampus and frontal cortex of the depressed rats [49]. Zeng et al. examined the antidepressant effects of Rosmarinic acid (RA), which is one of the polyphenol compounds found in a variety of herbs [50]. They induced depression-like behaviors in male C57BL/6 J mice by chronic intraperitoneal injection of CORT and performed daily oral application of RA for 21 consecutive days. They found that depression-like and anxiety-like behaviors, assessed with a forced swimming test (FST), tail suspension test (TST), splash test (ST), novelty-suppressed feeding test (NSFT), and sucrose preference test (SPT), were significantly alleviated by the application of RA [50]. Furthermore, it was revealed that hippocampal expression of pGR (glucocorticoid receptor), HSP90 (heat shock protein 90), FKBP51 (FK506 binding protein 51), and SGK-1 (recombinant serum/glucocorticoid regulated kinase 1) was decreased by treatment with RA while a density of Nissl bodies and the number of dendritic spines in the hippocampus were increased after RA treatment. Interestingly, expressions of BDNF, pTrkB, and pCREB were upregulated by RA [50].

Antineuroinflammatory and/or antidepressant effects from oleacein (OC), a rare compound obtained from olives, have been found [51]. In SH-SY5Y cells, the upregulation of BDNF was induced by OC application; however, the BDNF upregulation was canceled in the presence of an inhibitor for PI3K, or MEK (an upper molecule for ERK). Additionally, OC demonstrated a high affinity for the TrkB receptor and functioned as a TrkB agonist, leading to sustained activation of downstream signaling pathways. Interestingly, the administration of OC significantly improved depression-like behaviors in model mice caused by lipopolysaccharide (LPS). In addition, OC also decreased the mRNA levels of hippocampal pro-inflammatory cytokines including Il6, Il1β, and Tnfα in LPS mice [51].

It has been recognized that high-molecular-weight proteins including neurotrophins are not able to cross the BBB; thus, small molecules which mimic the actions of neurotrophins have been searched. Fukuyama et al. have summarized chemical constituents of neurotrophic compounds derived from Garcinia subelliptica, Magnolia ovobata, Phytolacca americana, Illicium species, and Viburnum species, and showed their neurite outgrowth and neuroprotective effects using cellular systems including PC12, primary cortical neurons, and MEB5 cells [52].

These results indicate significant upregulation of BDNF by a variety of natural compounds, resulting in antidepressant-like effects in vivo and in vitro. The interventions concerning a mechanism underlying the upregulation of BDNF expression by these compounds could provide a beneficial platform to seek other drug candidates.

## 4. BDNF Mimetics and Their Antidepressant Effects in Depression Models

It was reported that impaired learning and memory, and exploratory behavior detected in depressed rats after receiving unavoidable electric foot-shock were corrected by GSB-106, N-monosuccinyl-L-seryl-L-lysine hexamethylenediamide, which is a dipeptide mimetic of BDNF loop 4 [53]. To analyze the effectiveness of GSB-106 against depression, an animal study was performed using a depressive mouse model caused by 28-day social defeat stress with 21-day oral administration of GSB-106 after the stress [54]. Decreased locomotor activity and an impaired sucrose preference were restored by the administration of GSB-106. Remarkably, the antidepressant-like activity of GSB-106 assessed with FST was canceled by K252A, a blocker for Trk receptors, and by U73122, an inhibitor for PLC, suggesting probable contribution of TrkB/PLCγ signaling to the effects of GSB-106 [54]. Furthermore, male rats exhibited depression-like behavior and impaired memory after transient middle cerebral artery occlusion (MCAO), although GSB-106 administration completely reversed these depression-like behaviors and memory impairment [55]. Moreover, it was revealed that the decreased content of CREB in the hippocampus of MCAO animals was also repaired by GSB-106 administration [55].

Since BDNF plays a pivotal role in antidepressant effects observed in animal models, BDNF mimetics, which stimulate TrkB receptor, are promising candidates to treat depression. However, further comparative studies are required to evaluate the degree and duration of TrkB activation by these mimetics, compared to BDNF itself.

## 5. A Variety of Mechanisms Under the Influence of BDNF in Depression Models

Tiliwaerde et al. recently showed that GW043, which is a novel NMDA receptor modulator, has antidepressant effects by using an animal model [56]. They found that the activation of NMDAR and hippocampal long-term potentiation (LTP) were induced by GW043 in mice. Furthermore, depressive behaviors in rats caused by chronic unpredictable mild stress (CUMS) were reversed by GW043 treatment. Downregulation of BDNF and phosphorylation of mTOR in the hippocampus and prefrontal cortex of CUMS rats was reversed by GW043 [56]. Growing evidence has demonstrated the possible involvement of histone deacetylase 5 (HDAC5) in the pathogenesis of MDD. Meng et al. performed the overexpression of HDAC5 with adenoviral vectors in the dentate gyrus of mice and found that depression-like behaviors were evoked by the overexpression. Importantly, in addition to the improvement of depressive-like behaviors, the downregulated BDNF expression (mRNA and protein) in the hippocampus of mice overexpressing HDAC5 was restored by application of TMP269, a class IIA histone deacetylase inhibitor [57].

Recently, the effects of polygonatum sibiricum polysaccharides (PSPs) on microglia have been shown [58]. The depression-like behaviors (with SPT and FST) observed in mice with chronic restraint stress (CRS) were significantly improved by PSP treatment. In the prefrontal cortex of the depressed mice, it was revealed that PSP decreased activation of microglia and upregulated microglial BDNF levels in the prefrontal cortex and hippocampal neurogenesis, suggesting an involvement of the regulation of microglia in the effects of PSP [58]. Valenza et al. reported the responses of astrocytes and microglia to acute stress. In a rat model of post-traumatic stress disorder, an acute inescapable foot shock caused prompt astrocyte and microglia responses, NF-κB pathway activation, and an increase in the levels of IL-18 and TNF-α in the prefrontal cortex of vulnerable rats. Furthermore, significantly changed levels of glial proteins (S100B, CD11b, and CX43) and trophic factors (BDNF and FGF2) were also confirmed [59].

Interestingly, a recent study reported that patients with MDD displayed lower levels of BDNF in plasma astrocyte-derived extracellular vesicles (ADEVs) than healthy controls at baseline [60]. Importantly, BDNF levels in ADEVs were more stable than in plasma, suggesting that ADEVs are more suitable for biomarkers than plasma in depression [60].

As these findings indicate, in addition to neuronal population, it is possible that dysfunctions of glial cells, including astrocytes and microglia, are involved in the pathophysiology of depressive disorders. Thus, the biological responses of glial cells to BDNF mimetics are crucial, and further investigation into the mechanisms underlying glial contribution to neuronal functions and their roles in the pathophysiology of depression is essential.

## 6. Relationship Between BDNF/TrkB System and Schizophrenia

Zou et al. performed a network meta-analysis on different psychiatric disorders, and found that peripheral BDNF concentration in patients with MDD, bipolar disorder, obsessive–compulsive disorder, panic disorder, or schizophrenia is lower compared with that in controls, although patients with PTSD showed increased BDNF levels, suggesting BDNF is a valuable biomarker for the diagnosis of mental illnesses [61].

Schizophrenia, a severe and highly heritable psychiatric disorder, also exhibits abnormality in thinking and emotions, and has been suggested to be associated with altered BDNF function. Importantly, using the search terms (schizophrenia) AND (BDNF gene polymorphism) to find articles published in the last 5 years, Farcas et al. conducted a systematic search on Val66Met polymorphism of the BDNF gene and showed that the Met/Met genotype is associated with higher positive scores on the Positive and Negative Syndrome Scale (PANSS), reflecting disease severity, and with a history of suicide attempts [15]. Moreover, individuals with homozygous Met/Met exhibit lower levels of peripheral BDNF and worse cognitive ability [15]. Indeed, a recent systematic review of the literature on the differences in baseline levels of BDNF and a Val66Met polymorphism of BDNF in bipolar disorder and schizophrenia among responders and non-responders presents that higher levels of baseline BDNF and the BDNF Val/Val genotype may be present in responders to both pharmacological and non-pharmacological treatments [62].

Chen et al. reported schizophrenia-like behaviors in Bdnf-e6-/- mice (promoter VI mutant mice) when exposed to postnatal stress, although stress or the deficiency of Bdnf promoter alone did not cause behavioral abnormalities. These behavioral abnormalities were improved by RU-486 (an antagonist for corticosterone) or Ab4B19 (an agonistic antibody for TrkB), suggesting both environmental and genetic factors contributing to the pathogenesis of schizophrenia [16]. In addition to the BDNF gene, the expression of antisense RNA for the BDNF gene (BDNF-AS) has also been demonstrated to be involved in the vulnerability to psychiatric disorders, including depression, schizophrenia, and bipolar disorder [63]. As expected, a lot of studies suggest significant connections among specific polymorphisms of the BDNF-AS gene and these psychiatric disorders [63], reconfirming an importance of BDNF expression in the pathogenesis of psychiatric disorders including schizophrenia because of its role in the regulation of BDNF expression. A recent cross-sectional comparative study (208 healthy control subjects, 123 patients with mild cognitive impairment (MCI), and 123 patients with schizophrenia) also reported a lower concentration of plasma BDNF levels in patients with schizophrenia and MCI compared with those in healthy controls [64]. It was revealed that the cognitive functions (estimated by mini mental state examination) of patients with schizophrenia and individuals with MCI were lower than those in controls, and the lower cognitive activity showed a significant association with decreased plasma BDNF levels [64].

As shown, increasing evidence suggests that restoring the BDNF/TrkB system may help alleviate symptoms of schizophrenia. However, since schizophrenia exhibits distinct pathophysiological characteristics compared to depression, a mood disorder, further studies using schizophrenia models are needed to better understand the role of BDNF in its pathogenesis and potential therapeutic implications.

## 7. Contribution of BDNF/TrkB System in Antipsychotic Effects of Natural Compounds in Schizophrenia Models

As we will discuss in Section 8, 7,8-dihydroxyflavone (7,8 DHF), a flavonoid with significant neuroprotective effects in various animal models of neurodegeneration, has been extensively studied [65]. Discovered through high-throughput screening, 7,8-DHF was identified as a small molecule which is capable of crossing the BBB and selectively binding to TrkB [66]. Even in regard to schizophrenia models, the effects of 7,8 DHF have been examined. Han et al. showed possible involvement of downregulation of the BDNF/TrkB system in the abnormal neurodevelopment of offspring produced by polyriboinosinic-polyribocytidylic acid [poly(I:C)] [67]. In their system, adult offspring from pregnant animals which received poly(I:C) exhibited decreased BDNF/TrkB signaling and immunoreactivity of parvalbumin in the prefrontal cortex and hippocampus, in addition to PPI deficits, although supplementation of 7,8 DHF reversed all these abnormalities, including the downregulation of BDNF/TrkB signaling, reduced PV immunoreactivity, and abnormal behavior [67]. Using an animal maternal immune activation (MIA) model which was induced with polycytidylic acid (Poly-I:C) infection during pregnancy, the schizophrenia-like behaviors of the offspring were examined [68]. Fetal brains from MIA dams showed increased IL-6 and downregulation of Ntrk2 and glutamatergic/GABAergic neuronal markers. As expected, adult offspring from MIA dams exhibited anxiety-like behaviors. Interestingly, 7,8-DHF applied through drinking water for MIA dams could not inhibit the negative influence of MIA on behavioral disturbances in adult offspring although 7–8 DHF treatment reversed the expression of some glutamatergic (Grm5) and GABAergic (Gabra1) genes, implying an importance of the timing of potential interventional treatment [68]. When exposed to Poly I:C, cultured hippocampal neurons exhibited reduced cell viability and increased lipoperoxidation, and showed inflammatory responses including upregulation of the NFkB canonical pathway and decreased expression of BDNF [69]. Interestingly, omega-3 polyunsaturated fatty acids (n3 PUFAs) and clozapine, an atypical antipsychotic, inhibited the pro-inflammatory responses, although only n3 PUFAs prevented both neuronal cell death and the depletion of BDNF expression caused by exposure to Poly I:C [69]. Using an animal model for intrauterine growth retardation (IUGR) caused by maternal administration of thromboxane A2, we also found significant downregulation of TrkB in the cortex of IUGR rats at birth [29]. Interestingly, glutamate release triggered by BDNF was decreased in cultured IUGR cortical neurons where the decreased TrkB was still maintained, which were reversed after the transfection of human TrkB. It was reported that the downregulation of the BDNF/TrkB system, deficits in learning ability, and hippocampal synaptic plasticity of the schizophrenia-like animals were also rescued by 7,8-DHF [70]. MK-801-treated rats exhibited poor working learning ability (MWM test), decreased expression of BDNF, and deficits in hippocampal LTP, despite enhanced activation of TrkB, ERK1/2, CaMKII, CREB, and GluR1, with an improved synaptic plasticity and learning ability that were observed after the chronic 7,8-DHF treatment [70]. Using a mouse model of schizophrenia induced by ketamine, an NMDA antagonist, the effects of a polyphenolic flavonoid, silymarin, on disease-like symptoms were examined [71]. Preventive and curative effects of silymarin against hyperlocomotion, stereotypy, memory, and social impairments were observed and significant downregulation of BDNF, glutathione, superoxide-dismutase, and catalase in the prefrontal cortex, hippocampus, and striatum of mice that received the ketamine administration were all restored by silymarin [71]. Khalid et al. examined the antipsychotic effects of esculetin, a derivative of natural coumarin, against the schizophrenia-like behaviors of mice that received the chronic administration of ketamine [72]. They found improvements in the behavioral symptoms, oxidative stress, and status of neuroinflammation caused by ketamine. Further, they observed restoration of BDNF, which was decreased in the hippocampus, cortex, and striata by ketamine [72].

Ordinarily, both fingolimod (also named FTY-720) and siponimod, which are modulators for a sphingosine 1-phosphate receptor (S1PR), have been recognized as effective drugs to inhibit the progression and relapse of multiple sclerosis [73,74,75]. Recently, to identify molecular targets to reverse the decreased cognitive function of schizophrenia, Li et al. performed bioinformatics and computational analyses focusing on the pharmacological mechanisms underlying neuroprotective effects by both fingolimod and siponimod [76]. Interestingly, the regulations of the MAPK and PI3K/Akt signaling pathways involving TNF, IL1B, IL6, INS, BCL2, AKT1, and BDNF by fingolimod and the MAPK signaling pathways involving TNF, AKT1, and CASP3 (Caspase-3) by siponimod have been demonstrated [76].

As a genetic variation in CACNA1C (the alpha-1 subunit of CaV1.2, L-type voltage-dependent Ca^2+^ channels) is demonstrated to be a risk factor for both bipolar disorder and schizophrenia, using a Cacna1c+/− rat model [77], Tigaret et al. examined cognitive and synaptic phenotypes of the animal models and found synaptic and cognitive abnormalities in the rats, which were also rescued by LM22B-10, an agonist for the TrkB/TrkC receptor [17]. Recently, it was reported that LT-102, a novel potentiator of AMPAR, enhanced the hippocampal LTP and restored cognitive deficits in a schizophrenia-like animal model induced by phencyclidine [78]. LT-102 bound to AMPAR’s GluA2 subunit and induced upregulation of BDNF protein and the phosphorylation levels of GluA1 [78].

**Figure 2:**
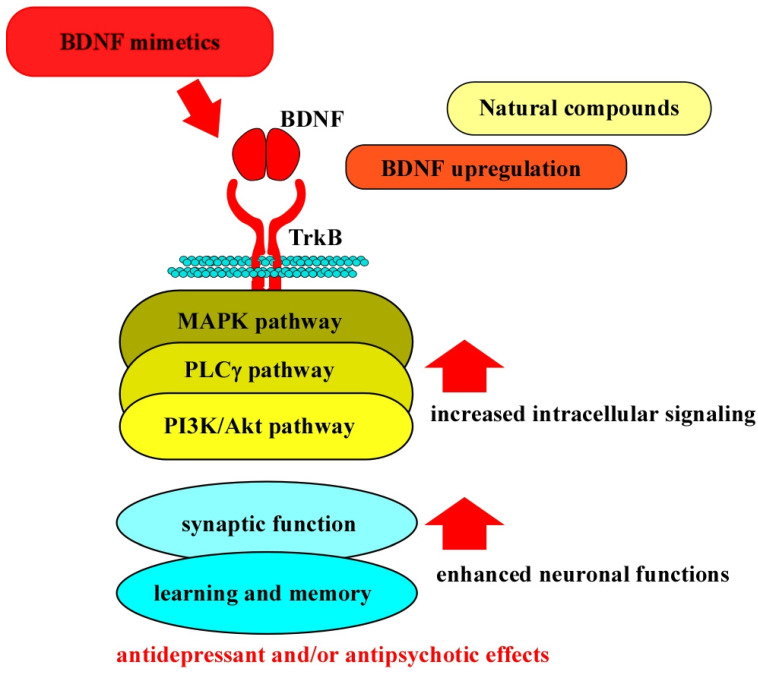
BDNF mimetics, natural compounds, and the BDNF/TrkB system. It is possible that the downregulation of the BDNF/TrkB system is involved in impaired neuronal function associated with psychiatric disorders, including depression and schizophrenia. Thus, BDNF mimetics, which stimulate TrkB, and natural compounds, which can increase the expression of endogenous BDNF (and/or TrkB), can restore the activation status of intracellular signaling (MAPK-, PLCγ-, and PI3K/Akt-pathways), leading to the potentiation of synaptic function, and learning and memory in patients.

As shown in these sections concerning psychiatric disorders, boosting BDNF function through its specific receptor TrkB is indeed effective in improving symptoms of depression and schizophrenia, as judged by the results from using in vivo and in vitro models (see Figure 2). Furthermore, altered levels of biomarkers, including BDNF and/or downstream signaling molecules, for mental illnesses clearly suggest the promising potential of BDNF mimetics. However, further information concerning the actions of BDNF mimetics, including the duration of TrkB stimulation and potential side effects at the cellular level, should be obtained before clinical applications as excess BDNF/TrkB activation may cause adverse reactions, resulting in abnormal synaptic plasticity. However, some different symptoms between depression and schizophrenia exist; a more detailed analysis of the contribution of the BDNF/TrkB system and its signaling pathway, using in vivo and in vitro models, is required.

## 8. The Therapeutic Potential of BDNF Mimetics in Alzheimer’s Disease (AD)

### 8.1. The Role of BDNF in AD

**Figure 3:**
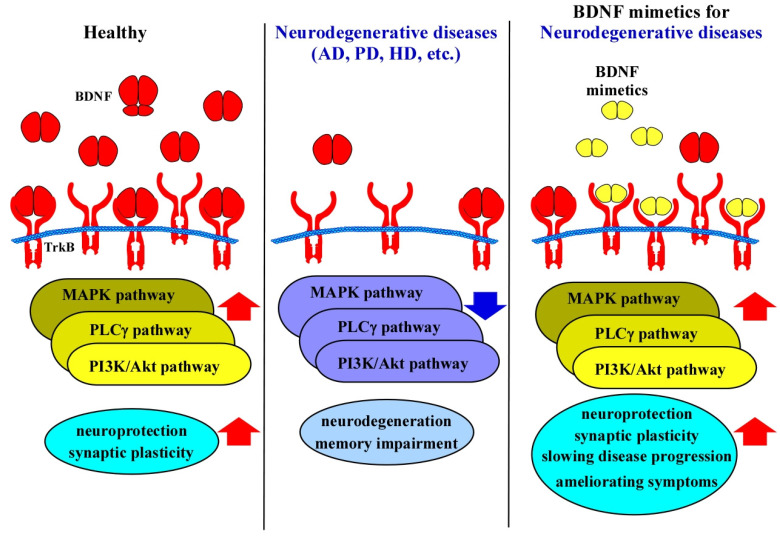
The role of the BDNF/TrkB system in neurodegenerative diseases and the therapeutic potential of BDNF mimetics for these conditions. BDNF exerts its effects primarily by binding to TrkB receptors, initiating downstream signaling pathways such as PI3K-Akt, MAPK-ERK, and PLCγ, which are critical for neuroprotection, synaptic development, and plasticity. In neurodegenerative disorders such as AD, PD, and HD, reduced levels of BDNF and/or TrkB impair intracellular signaling, leading to synaptic loss and neuronal atrophy—key features of neurodegeneration. BDNF mimetics, which are designed to replicate the neurotrophic and neuroprotective functions of BDNF by activating TrkB, offer an attractive alternative for treatment.

Several studies have demonstrated that BDNF levels are significantly reduced in the brains of AD patients, particularly in regions associated with memory, such as the hippocampus [79,80]. This decline is thought to exacerbate neuronal loss and cognitive decline, making BDNF signaling a critical therapeutic target (Figure 3). However, delivering BDNF itself as a treatment is challenging due to its large size and poor BBB permeability [81]. Consequently, the development of BDNF mimetics, which can circumvent these limitations, has garnered increasing attention.

As shown in Figure 1, the activation of TrkB initiates several intracellular cascades, including the PI3K/Akt, MAPK, and PLCγ pathways, all of which are implicated in cellular survival, synaptic growth, and plasticity. In AD, disruption of these pathways contributes to neurodegeneration [21]. Reduced levels of BDNF impair TrkB signaling, leading to synaptic loss and neuronal atrophy, which are hallmarks of AD pathology.

Given that synaptic dysfunction is one of the earliest signs of AD, strategies aimed at enhancing TrkB signaling could provide significant therapeutic benefits. While direct administration of BDNF has shown neuroprotective effects in preclinical models [81,82,83], its therapeutic application in humans remains impractical due to delivery challenges and a short half-life. As a result, BDNF mimetics, which are designed to replicate the neurotrophic and neuroprotective functions of BDNF by activating TrkB, offer an attractive alternative for AD treatment.

### 8.2. Challenges in Direct BDNF Therapy

The biological hurdles to directly using BDNF as a therapeutic agent for AD were recognized early on. Despite its promising neuroprotective properties, BDNF is a large protein (27 kDa noncovalently linked homodimer) with poor pharmacokinetic properties when administered systemically [81]. Intranasal delivery of BDNF, while bypassing the BBB, still faces challenges related to achieving therapeutic concentrations in the brain [84]. Furthermore, the short half-life of BDNF in circulation, combined with the need for repeated dosing, makes it a less viable long-term treatment option [85]. In addition, there are concerns regarding the specificity and safety of direct BDNF therapy. High concentrations of exogenous BDNF may lead to non-specific activation of not only TrkB but also other receptors, such as p75NTR, which may induce pro-apoptotic signals under certain pathological conditions [21]. These limitations highlighted the need to develop alternative strategies, specifically the use of small-molecule mimetics that can selectively target TrkB receptors while overcoming the pharmacological constraints associated with the BDNF protein.

### 8.3. Small-Molecule BDNF Mimetics: Early Efforts

The first generation of BDNF mimetics focused on small molecules with an ability to activate TrkB, either by mimicking BDNF binding or by inducing receptor dimerization and autophosphorylation. One of the most studied early candidates is 7,8-DHF, a flavonoid that has demonstrated significant neuroprotective effects in various animal models of neurodegeneration [65]. Discovered through high-throughput screening, 7,8-DHF was identified as a small molecule which is capable of crossing the BBB and selectively activating TrkB [66]. In preclinical studies, 7,8-DHF has been shown to exert multiple beneficial effects, including the restoration of synaptic plasticity, the reduction of Aβ deposition, and the mitigation of cognitive deficits in AD models [19,20,22]. By activating TrkB, 7,8-DHF triggers downstream signaling cascades such as the PI3K/Akt, MAPK/ERK, and PLCγ pathways, thereby promoting neuronal survival, reducing apoptosis, and enhancing synaptic strength—processes that are severely compromised in AD [22]. Its ability to cross the BBB and engage TrkB with relatively high specificity made it an attractive candidate for further development. Another example of the first generation of BDNF mimetics is LM22A-4, a small molecule that was specifically designed to target the extracellular domain of TrkB, thereby selectively activating TrkB without engaging the p75NTR or other neurotrophic receptors [86]. LM22A-4 demonstrated promising neuroprotective properties in animal models, including the promotion of neuronal survival, enhanced synaptic plasticity, and improved cognitive outcomes in rodent models of neurodegeneration including AD [87,88]. However, despite these promising preclinical results, 7,8-DHF and LM22A-4 face some limitations in their pharmacokinetic properties, including rapid metabolism and poor oral bioavailability [65]. Efforts have been made to improve their stability and effectiveness, leading to the synthesis of structural analogs with enhanced pharmacological profiles. Despite these challenges, the first generation of BDNF mimetics remain foundational molecules in the field of BDNF mimetic development, demonstrating the feasibility of small-molecule approaches to induce TrkB activation.

### 8.4. Next-Generation BDNF Mimetics: Improved Selectivity and Potency

The limitations of first-generation mimetics such as 7,8-DHF and LM22A-4 spurred the development of second-generation compounds with improved pharmacokinetics, BBB permeability, and receptor selectivity. For example, Chen et al. developed a prodrug of 7,8-DHF named R13 by modifying the catechol group with a carbamate moiety, which resulted in significantly improved drug-like characteristics [89]. Chronic oral administration of R13 activated TrkB signaling and reduced Aβ deposition in 5xFAD mice, thereby inhibiting the pathological cleavage of APP and tau mediated by δ-secretase. Furthermore, R13 mitigated hippocampal synapse loss and alleviated memory impairments in a dose-dependent manner [89]. Importantly, R13 appears to exhibit a more favorable pharmacokinetic profile compared to 7,8-DHF, with better stability and specificity for TrkB. Another study further optimized 7,8-DHF, producing a highly stable CF3CN derivative that demonstrates TrkB agonist activity in cellular assays with an EC50 of approximately 26.4 nM [90]. Chronic oral administration of CF3CN exhibited promising therapeutic effects in a 5xFAD mouse model [90].

Beyond small molecules, the development of BDNF mimetic peptides has gained traction in recent years. Peptide-based mimetics are designed to interact directly with the ligand-binding domain of TrkB, effectively replicating BDNF’s action but in a smaller, more manageable form. For example, the peptide GSB-214 was engineered to selectively activate TrkB and promote neurotrophic signaling in animal models [91]. Importantly, GSB-214 has shown promise in preventing memory impairment in AD-model rats [92]. Peptide-based approaches have certain advantages, including high specificity for the target receptor and minimal off-target effects. However, they are often limited by poor oral bioavailability and susceptibility to enzymatic degradation. Researchers are actively working on overcoming these obstacles by modifying peptide structures, enhancing their stability, and developing novel delivery methods to improve their therapeutic potential.

### 8.5. Preclinical and Clinical Development

While the majority of research on BDNF mimetics remains in the preclinical stage, there has been significant progress in moving promising candidates toward a clinical trial. For instance, one study mentions that R13 has entered a phase 1 clinical trial as a potential treatment for AD, though the result from the trial is still pending [93].

Ongoing preclinical studies continue to evaluate the safety, efficacy, and optimal dosing of second-generation mimetics as well as peptide-based compounds. Recently, Liao et al. demonstrated that the combination therapy of the optimized δ-secretase inhibitor (#11a) and CF3CN exhibited strong pharmacokinetic and pharmacodynamic properties in vivo, efficiently suppressing δ-secretase activity in the brain of a 3xTg AD mouse model [94]. Oral administration of both #11a and CF3CN substantially delayed AD progression and improved cognitive functions, with their combination producing the greatest therapeutic effect [94].

A key challenge in the clinical development of BDNF mimetics is demonstrating their long-term efficacy and safety in human patients. Neurodegenerative diseases like AD progress slowly, making it essential to assess whether these mimetics can provide sustained neuroprotective effects over extended periods. Moreover, the potential for TrkB overactivation, which could lead to unwanted side effects such as abnormal cell proliferation, must be carefully monitored in clinical studies.

## 9. The Therapeutic Potential of BDNF Mimetics in Parkinson’s Disease (PD) and Huntington’s Disease (HD)

### 9.1. The Role of BDNF in PD

While both genetic and environmental factors contribute to PD etiology, a key area of interest has been the neurotrophic support mechanisms, especially the role of BDNF. Several studies have demonstrated that BDNF levels and signaling are significantly altered in PD, suggesting that impaired BDNF function may exacerbate dopaminergic neuronal degeneration [23].

BDNF supports dopaminergic neuron health through activation of the TrkB receptor, promoting signaling pathways that counteract cellular stress and apoptosis [95]. In PD, both endogenous BDNF expression and TrkB receptor activation disappeared in the substantia nigra, resulting in reducing neuroprotection and allowing oxidative stress and mitochondrial dysfunction to induce neuronal death (Figure 3) [96]. Studies using animal models of PD have shown that BDNF administration can restore dopaminergic neuron populations and improve motor function, underscoring its neuroprotective potential [97,98]. Additionally, BDNF’s role in synaptic plasticity is crucial in maintaining functional neural circuits that are compromised by the progressive neuronal loss in PD [5].

BDNF’s involvement in PD is further complicated by various mechanisms contributing to its dysregulation. Genetic factors, including single-nucleotide polymorphism in the BDNF gene, have been linked to reduced BDNF expression in PD patients [99]. Additionally, epigenetic factors, such as DNA methylation and histone modification, can alter BDNF expression [100]. The relationship between alpha-synuclein, a protein implicated in PD pathology, and BDNF signaling has also been of interest. Studies suggest that alpha-synuclein accumulation may impair BDNF trafficking and reduce its availability to dopaminergic neurons [96,101]. Moreover, inflammatory processes common in PD can interfere with BDNF expression and signaling, further hindering its neuroprotective functions [102].

The potential to harness BDNF for therapeutic interventions in PD is promising but presents numerous challenges. Direct BDNF administration has shown positive effects in preclinical models [97,98], yet its therapeutic application in humans is limited by the protein’s poor BBB permeability and rapid degradation. Novel delivery methods, such as nanocarriers, and small molecules that mimic BDNF activity, are under investigation [5]. Small molecules that activate TrkB or modulate endogenous BDNF levels represent another promising area for PD therapeutics, although safety and efficacy in human trials remain to be fully established.

### 9.2. BDNF Mimetics in PD

One of the most well-studied BDNF mimetics in PD is also 7,8-DHF. Preclinical studies have demonstrated that 7,8-DHF can prevent dopaminergic neuron loss, reduce oxidative stress, and improve motor function in animal models of PD [103,104,105]. For example, treatment with 7,8-DHF in rodent models of PD resulted in significant preservation of dopaminergic neurons in the substantia nigra and increased levels of striatal dopamine, translating into improved motor performance [106]. The neuroprotective effects of 7,8-DHF are attributed to its ability to activate key survival pathways, including the PI3K/Akt and ERK pathways, which are essential for reducing neuronal apoptosis and promoting synaptic plasticity [5]. Additionally, 7,8-DHF has been found to attenuate neuroinflammation, which is increasingly recognized as a contributing factor in PD pathology. By reducing microglial activation and the release of pro-inflammatory cytokines [107], 7,8-DHF may offer a dual benefit by protecting neurons and reducing neuroinflammatory damage. Other small-molecule TrkB agonists are also being actively investigated due to their potential for oral administration and ability to cross the BBB [108]. CF3CN has shown efficacy in promoting TrkB activation, inhibiting δ-secretase, and increasing TH-positive dopaminergic neurons in MPTP-induced human SNCA transgenic PD mice [109]. These agents are attractive candidates because they can be synthesized with structural modifications that enhance their specificity for TrkB, thereby reducing the risk of side effects associated with non-specific neurotrophin receptor activation.

Beyond small-molecule TrkB agonists, other classes of BDNF mimetics are being explored, including peptidomimetics—short peptides or peptide-like molecules designed to mimic specific regions of the BDNF protein that interact with the TrkB. These peptidomimetics are engineered for enhanced receptor specificity, minimizing off-target effects and reducing the risk of adverse reactions. Indeed, the peptide compound GSB-214 has demonstrated TrkB agonist activity, promoting neuroprotection and ameliorating motor dysfunction in a 6-OHDA-induced rat model of PD [110].

### 9.3. The Role of BDNF in HD

HD is a hereditary neurodegenerative disorder characterized by progressive motor dysfunction, cognitive decline, and psychiatric disturbances. This disease is caused by an expansion of CAG trinucleotide repeats in the huntingtin (HTT) gene, leading to the production of a mutant huntingtin protein (mHTT) that is prone to aggregation. These aggregates are toxic to neurons, particularly in the striatum and cortex, resulting in widespread neuronal dysfunction and cell death. Despite the well-established genetic basis of HD, the precise molecular mechanisms that contribute to neurodegeneration remain incompletely understood. However, evidence increasingly points to deficiencies in neurotrophic support, especially by BDNF, as a crucial factor in HD pathophysiology [24].

The hippocampus, cortex, and striatum—regions critically affected in HD—are particularly reliant on BDNF signaling for their function and structural integrity [24]. However, in HD, there is a marked reduction in BDNF levels, which has been implicated as a significant contributor to disease progression (Figure 3) [111,112]. The reduction in BDNF levels in HD is thought to occur through multiple mechanisms. One of the primary contributors is the dysregulation of transcriptional machinery caused by mHTT. The transcriptional repressor element-1 silencing transcription factor (REST) functions as a corepressor, inhibiting the expression of its target genes, including BDNF. The wild-type Htt protein, located in the cytosol, interacts with REST, thereby preventing its translocation into the nucleus and consequently promoting the expression of its target genes through derepression. In contrast, mHtt exhibits a reduced affinity for REST, which facilitates REST’s nuclear localization and results in the suppression of its target genes, such as BDNF [113,114]. As a result, the synthesis of BDNF is significantly impaired. Furthermore, mHTT has been shown to disrupt axonal transport, thereby hindering the delivery of BDNF from the cortical neurons, where it is synthesized, to the striatal neurons, where it is required for cellular health [115]. This impaired transport mechanism exacerbates the striatal atrophy observed in HD patients. Additionally, BDNF signaling is compromised at the receptor level in HD (Figure 3). A previous study has demonstrated that TrkB receptor expression is decreased in the brains of HD patients [116], which further impairs the neurotrophic support needed to counteract neurodegenerative processes. This reduction in both BDNF availability and TrkB receptor activity creates a vicious cycle of neuronal vulnerability, synaptic dysfunction, and cell death. The downstream signaling cascades activated by the TrkB, PI3K/Akt, and MAPK pathways are essential for promoting cell survival and neuroprotection. In HD, the downregulation of the BDNF/TrkB system disrupts these protective pathways, contributing to the accelerated neurodegeneration observed in affected brain regions.

The interplay between BDNF deficiency and HD pathology is further supported by animal model studies. Importantly, genetic or pharmacological interventions that increase BDNF levels have been shown to alleviate some of the motor and cognitive deficits in these models [117,118,119], suggesting that enhancing BDNF signaling could be a viable therapeutic strategy. For instance, the overexpression of BDNF in HD mouse models has been reported to delay disease onset and improve motor function, providing compelling evidence for the neuroprotective role of BDNF [117].

Collectively, these findings underscore the critical role of BDNF in the pathogenesis of HD. The loss of BDNF and the resulting impairments in TrkB-mediated signaling contribute to neuronal dysfunction, synaptic deficits, and neurodegeneration, which are hallmarks of HD. The therapeutic potential of strategies aimed at restoring BDNF levels or mimicking its activity has garnered significant interest. Given the central role of BDNF in neuronal survival and plasticity, targeted interventions to enhance the BDNF/TrkB system could provide a promising avenue for slowing disease progression and ameliorating symptoms in HD patients.

### 9.4. BDNF Mimetics in HD

Several classes of BDNF mimetics have been developed, each with distinct mechanisms of action aimed at either directly activating TrkB or enhancing TrkB-mediated signaling indirectly. As expected, 7,8-DHF has shown considerable promise in preclinical studies in HD-model mice [120,121,122]. By binding to TrkB, 7,8-DHF activates the PI3K/Akt and MAPK pathways, enhancing neuronal survival and plasticity while also promoting synaptic function in HD [120,121,122]. Another mimetic, LM22A-4, has also demonstrated efficacy in mitigating HD pathology [123]. In R6/2 HD mice, systemic administration of LM22A-4 led to the activation of TrkB, along with the activation of downstream pathways, including Akt, PLCγ, and CREB. Additionally, it reduced intranuclear HTT aggregates within the striatum and cortex, suppressed microglial activation in the striatum, and protected striatal neurons from degeneration associated with HD [86]. Moreover, LM22A-4 preserved dendritic spine density and enhanced motor function in both R6/2 and BACHD mouse models [86].

In addition to directly targeting TrkB, some small molecules function via modulating endogenous BDNF levels. Compounds that enhance the expression or release of BDNF from neurons can potentially address the underlying deficit observed in HD. HDAC inhibitors, for example, have been shown to increase BDNF levels by promoting chromatin relaxation, thereby facilitating gene transcription [124,125]. The use of HDAC inhibitors like suberoylanilide hydroxamic acid (SAHA) and CKD-504 in HD models has demonstrated neuroprotective effects, partly attributable to increased BDNF expression [126,127]. Furthermore, certain natural compounds have been identified as potential BDNF mimetics due to their ability to enhance TrkB-mediated signaling indirectly. For example, curcumin, an active ingredient in turmeric, has shown neuroprotective properties in R6/2 HD mice, partly by upregulating BDNF expression and activating TrkB signaling pathways [128]. The compound not only possesses neurotrophic activity but also exhibits anti-inflammatory and antioxidant properties, which may further mitigate the neurodegenerative processes in HD [129].

## 10. Conclusions and Future Directions

The exploration of BDNF mimetics as therapeutic agents has opened new avenues for the treatment of neuropsychiatric and neurodegenerative disorders. The dysregulation of the BDNF/TrkB system is a shared pathological hallmark across a wide range of conditions, including depression, schizophrenia, AD, PD, and HD. Consequently, restoring TrkB signaling has emerged as a promising strategy for mitigating the neuropsychiatric or neurodegenerative processes that are characteristic of these disorders.

However, despite encouraging preclinical evidence, there are several challenges to translating the therapeutic potential of BDNF mimetics into effective clinical treatments. One of the most significant barriers is the pharmacokinetic profile of current BDNF mimetics. For example, while 7,8-DHF has shown efficacy in animal models, its bioavailability and stability in humans remain uncertain. Ensuring that these compounds can achieve therapeutic concentrations in the brain without rapid degradation or off-target effects is crucial. Additionally, the ability of BDNF mimetics to effectively penetrate the BBB in human patients remains a significant hurdle. Advances in drug delivery systems, such as nanoparticle carriers, or prodrug formulations, may enhance the bioavailability of these compounds, thereby increasing their therapeutic potential. Various strategies are being investigated to improve the delivery of BDNF across the BBB. These include approaches such as the “Trojan Horse” delivery method [130], the use of BBB modulators [131], magnetic nanocarriers [132], and ultrasound combined with microbubbles [81]. Each of these methods has shown varying levels of success in facilitating the transport of therapeutic agents into the brain. For instance, as a molecular Trojan horse strategy, BDNF was conjugated to a monoclonal antibody (MAb) targeting the transferrin receptor, an endogenous receptor involved in BBB transport [133]. Intravenous administration of this BDNF-MAb conjugate successfully crossed the BBB and improved neurobehavioral outcomes in a rat stroke model [133].

Moreover, a deeper understanding of disease-specific alterations in the BDNF/TrkB system is essential to optimize the use of BDNF mimetics. The patterns of BDNF dysregulation in AD, PD, and HD are complex and may vary across disease stages and brain regions. Precision medicine approaches that tailor BDNF-based therapies to the specific molecular profiles of individual patients could enhance the efficacy of these treatments. This would involve the integration of biomarkers such as cerebrospinal fluid BDNF levels, neuroimaging of TrkB receptor expression, or genetic profiling to predict patient responses to BDNF mimetics. Furthermore, investigating the temporal and spatial dynamics of TrkB activation at the cellular and circuit levels in both physiological and pathological conditions could uncover new therapeutic opportunities. Advanced tools such as single-cell RNA sequencing, spatial transcriptomics, and proteomics should be utilized to identify cell-type-specific roles of the BDNF/TrkB system across diverse neurological diseases. Additionally, more robust preclinical and clinical models are needed to evaluate the therapeutic efficacy of BDNF/TrkB modulators. Animal models that recapitulate the complexity and heterogeneity of human neurological diseases are crucial for this purpose. Parallelly, advancements in organoid and organ-on-chip technologies could facilitate the development of patient-specific models to test BDNF/TrkB-targeted therapies in a personalized manner.

It is also crucial to consider the potential benefits of combination therapies. Given the multifactorial nature of neuropsychiatric and neurodegenerative disorders, targeting the BDNF/TrkB system alone may not be sufficient to achieve robust clinical benefits. Combining BDNF mimetics with established therapies, such as selective serotonin reuptake inhibitors (SSRIs) for depression or anti-Aβ treatment for AD, could enhance therapeutic outcomes. Synergistic effects might be achieved by simultaneously addressing neurotransmitter imbalances, neuroinflammation, and oxidative stress alongside neurotrophic support. Furthermore, interventions like exercise, cognitive training, or dietary modifications known to naturally upregulate BDNF expression could complement the pharmacological effects of BDNF mimetics, offering a more holistic treatment strategy.

We described several small molecules which have been reported to act as TrkB agonists in this review; however, Boltaev et al. demonstrated the contradicting findings, emphasizing the need for cautious interpretation of experimental results involving these compounds [134]. In the study, they designed a series of assays to quantitatively assess TrkB activation and downstream signaling events in cells exposed to these reported BDNF agonists. Their analysis revealed that, unlike BDNF and other neurotrophic factors that activate TrkB, these compounds, including 7,8-DHF and LM22A-4, failed to stimulate critical components of TrkB signaling [134]. Therefore, we have to bear in mind that the development of reliable TrkB pharmacological agonists with robust activity that translates between in vivo and in vitro systems still remains an ongoing and inherent challenge.

In conclusion, BDNF mimetics represent a promising frontier in the treatment of neuropsychiatric and neurodegenerative disorders, offering the potential to address the neurotrophic deficits that underlie these conditions. While significant challenges remain, advances in drug delivery systems, selective receptor targeting, and personalized medicine approaches could pave the way for the successful translation of BDNF mimetics into clinical practice. Future research must continue to unravel the complexities of TrkB signaling in the brain and develop innovative strategies to harness its therapeutic potential. If these challenges can be overcome, BDNF mimetics may offer not just symptomatic relief but also disease-modifying benefits, ultimately improving the quality of life of patients suffering from these debilitating conditions.

## Data Availability

No new data were created or reported in this review article.
